# Social innovation in health: strengthening Community Systems for Universal Health Coverage in rural areas

**DOI:** 10.1186/s12889-022-14451-8

**Published:** 2023-01-09

**Authors:** Lindi van Niekerk, Martha Milena Bautista-Gomez, Barwani Khaura Msiska, Jana Deborah B. Mier-Alpaño, Arturo M. Ongkeko, Lenore Manderson

**Affiliations:** 1 London School of Hygiene and Tropical Medicine, London, UK; 2 Centro Internacional de Entrenamiento e Investigaciones Médicas (CIDEIM), Cali, Colombia; 3 Universidad Icesi, Cali, Colombia; 4 College of Medicine, University of Malawi, Blantyre, Malawi; 5 College of Medicine, University of the Philippines Manila, Manila, Philippines; 6 National Institutes of Health, College of Medicine, University of the Philippines Manila, Manila, Philippines; 7 University of the Witwatersrand, Johannesburg, South Africa

**Keywords:** Social innovation in health, Community empowerment, Agency, Universal health coverage

## Abstract

**Background:**

In seeking the attainment of Universal Health Coverage (UHC), there has been a renewed emphasis on the role of communities. This article focuses on social innovation and whether this concept holds promise to enhance equity in health services to achieve UHC and serve as a process to enhance community engagement, participation, and agency.

**Methods:**

A cross-country case study methodology was adopted to analyze three social innovations in health in three low- and middle-income countries (LMICs): Philippines, Malawi, and Colombia. Qualitative methods were used in data collection, and a cross-case analysis was conducted with the aid of a simplified version of the conceptual framework on social innovation as proposed by Cajaiba-Santana. This framework proposes four dimensions of social innovation as a process at different levels of action: the actors responsible for the idea, the new idea, the role of the institutional environment, and the resultant changes in the health and social system.

**Results:**

The study found that each of the three social innovation case studies was based on developing community capacities to achieve health through community co-learning, leadership, and accountability.

The process was dependent on catalytic agents, creating a space for innovation within the institutional context. In so doing, these agents challenged the prevailing power dynamics by providing the communities with respect and the opportunity to participate equally in creating and implementing programs. In this way, communities were empowered; they were not simply participants but became active agents in conceptualizing, implementing, monitoring, and sustaining the social innovation initiatives.

**Conclusion:**

The study has illustrated how three creative social innovation approaches improved access and quality of health services for vulnerable rural populations and increased agency among the intervention communities. The processes facilitated empowerment, which in turn supported the sustained strengthening of the community system and the achievement of community goals in the domain of health and beyond.

## Background

In recent times the conceptualization of health systems has evolved, from initially being regarded through a narrow, reductionist lens as comprised of ‘system hardware’ components, to include a greater awareness of and sensitivity to the role of human choice, ingenuity and ‘system software’ such as relationships, power and trust [1, 2]. In seeking the attainment of the Sustainable Development Goal target on Universal Health Coverage (UHC; SDG 3.8), the role of citizens and their communities has been re-emphasized. UHC is defined and aims at ensuring as all people have access to the health services they need, when and where they need them, without financial hardship. It includes the full range of essential health services, from health promotion to prevention, treatment, rehabilitation, and palliative care [3]. A cross-case review by Allotey and colleagues [4] states community participation and engagement “as key towards making the universality of health care possible,” especially for marginalized and previously excluded population groups.

Yet, despite positive phrases used in global policies and international guidelines emphasizing the value of ‘the participation of individuals, citizens and communities in the development and implementation of policies and plans [5] and the importance of mechanisms to ‘voice their needs and so influence the way care is funded, planned and provided ’[6], in reality, community engagement remains mostly a top-down prescription. At best, care is ‘co-produced’ under the guidance of an external expert and is not fully owned and led by communities. The World Health Organization defines community participation as occurring when people are enabled to become actively and genuinely involved in defining the issues of concern to them” [7]. A systematic review by George et al. [8] aimed to identify the extent to which participation of community health workers occurred across the full continuum, from identifying issues, to designing interventions, implementing these interventions, managing the necessary resources, and monitoring and evaluating the outcomes. In this review, the authors found that full participation was still very limited; from 260 health systems research studies reviewed, only four studies illustrated community involvement across the full continuum. Methodologies such as Participatory Appraisal or Participatory Action Research have been designed to overcome these problems by encouraging the involvement of community members in the discovery, planning, and implementation process [9], but despite their popularity, these methodologies are often ineffective or subverted [10, 11, 12, 13]. Furthermore, development critics such as Escobar [13] and Norber-Hodge [14] claim that all development methodologies coerce people to engage in a process initiated by outsiders; even participatory development interventions usurp community autonomy. There is some value in this statement as although community participation has been upheld since the 1978 Alma Ata declaration, it has not yet become an embedded and sustained phenomena in health systems. A reason for this thus could be as the authors above point out, the initiatiation of community participation processes have been done by external agencies and it has failed to give communities the opportunity to be the initiators or custodians of the process themselves, based on their needs and goals they wish to attain.

Haldane et al .[15] cautions further that empowerment is frequently perceived as an natural outcome of community participation, but often clear measurements of impact and empowerment require sustained engagement over time, which in turn are dependent on program sustainability. Although Haldane et al. [15] report evidence of community engagement as having a positive impact on health, they raise questions of how this be translated to a sustained reality at grassroots level. To answer this, we focus on social innovation, and consider whether this approach holds promise both to enhance equity in health services to achieve Universal Health Coverage, and to provide greater opportunities for community involvement and empowerment in health attainment.

**Fig. 1:**
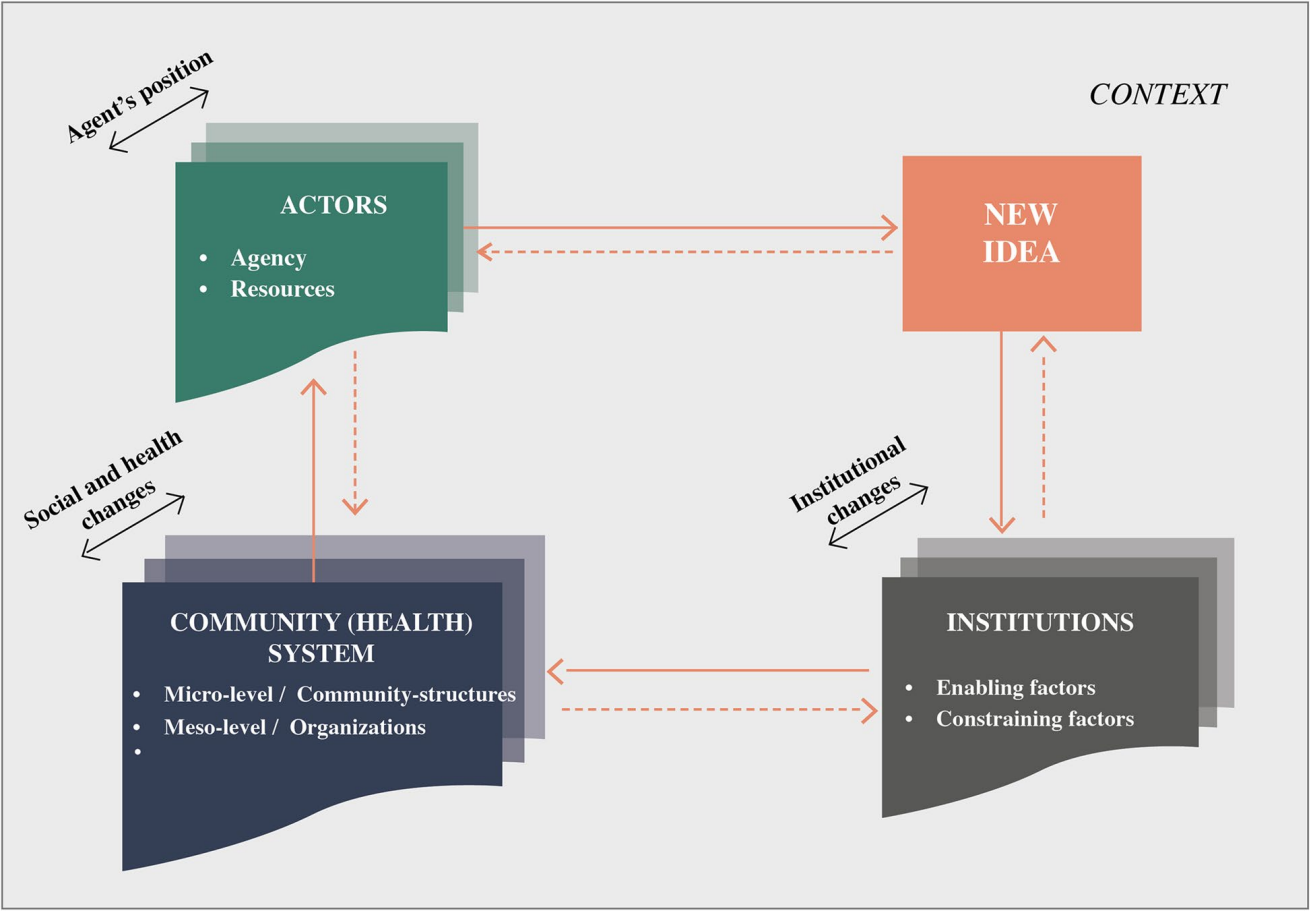
Social innovation process framework (modified) [20]

Social innovation has been hailed as a new way of achieving change in complex problem domains, especially those with convoluted overlaps in authority and multiple actors and institutions operating at varied scales [16]. Complex problems, like those faced by health systems, require more than mere improvements, tweaks, or controlled interventions. Rather, complexity demands adaptative solutions that can address the root causes of the problem while absorbing ongoing shocks [17]. Moulaert [18] defines three goals which social innovations seek to achieve: meeting unmet human needs (often neglected); raising participation levels among marginalized groups; and empowering people through greater access to resources and increased social and political capacities. Westley and Antandze [19] articulate another goal of social innovation, that of changing the underlying and internalized institutions responsible for the problem in the first place – “social innovation is a complex process of introducing new products, processes or programs that profoundly change the basic routines, resources and authority flows or beliefs of the social system in which the innovation occurs.” Cajaiba-Santana [20] defines social innovation as the collective, intentional, and goal-oriented creation of new practices aiming at social change. According to this author, social innovation takes place when a new idea establishes a different way of thinking and acting that changes existing paradigms, and this process is interactively influenced by both agents and social structures (See Fig. 1). This process implies that agents actively and reflexively interact with their social context, transforming and being transformed by it, as they promote social change through social innovation [20].

Social innovation does not always result in empowerment; the extent to which it does so depends on the paradigm adopted – a technocratic or a democratic paradigm of social innovation [21]. As stated by Montgomery, the technocratic paradigm of social innovation is underpinned by faith in experts (hero entrepreneurs) developing solutions to achieve efficiency, scale, and a return on investment [21]. Although ‘social capital building’ and ‘community empowerment’ are stated as objectives, this is tokenistic insofar as power distribution remains vertical, with the underlying political objective of reinforcing a neoliberal political agenda. In contrast, empowerment entails extending people’s capacity to make choices, to act and to bring about change. A lack of empowerment stems from inequalities in access to ‘opportunities and rewards for different social positions or status within a group’ [22]. The counter or democratic paradigm of social innovation thus embraces the horizontal distribution of power, and through this, seeks to establish greater inclusiveness, the participation of unlikely actors, and the mobilization of communities whose voices are not heard [21, 23]. The outcome of solutions arising from this democratic paradigm goes beyond impact in a specific social domain and results in creative transformations of social relationships that lead to shifts in power dynamics and, consequently, empowerment [21]. Ibrahim [24] demonstrates how social innovation can result in empowerment through expanding people’s agency. Especially in resource constrained settings, empowerment often occurs as result of people’s expanded individual and collective agency, leading to the re-organization and re-distribution of social capital along with other already existing resources at both a communal and societal level. Social innovation operates as a conversion factor to turn people’s needsand aspirations to become met needs or achieved goals [25].

The purpose of this article is to explore the outcome of three social innovations in health in rural community systems in Malawi, Philippines and Colombia, and consider whether and how the agency and empowerment of community members are achieved by these initiatives. We consider the various approaches taken to enhance healthcare for rural low-resource populations, the role of initiating actors, the influencing institutional factors, and the effects that the innovation had on the community as a whole, including in relation to health. We conclude by describing the implications social innovation may hold for the extension of UHC, and whether social innovation could help extend beyond tokenism our current notion of people-centeredness and community participation.

## Methods

### Study design and context

Given the limited knowledge about social innovation in health within low- and middle-income countries (LMICs), qualitative case study research was selected as an appropriate methodology. A key feature of case study research is its exploratory and explanatory potential. It enables the description and analysis of phenomena occurring in ‘open systems’ where context is not controlled, and variables interact in changing ways over time [26]. Case studies are regarded as a key source of information in exploring the effectiveness of social and cultural strategies in social innovation research [27].

We conducted data collection to produce individual social innovation case studies, and subsequently conducted a secondary cross case analyses on the data to assess, in particular, the influence of these social innovation on UHC. To identify cases for this analysis, 38 case studies from the TDR Social Innovations in Health Initiative repository were reviewed (www.socialinnovationinhealth,org). This repository is comprised of case studies, which were included following six public crowdsourcing contests across LMIC countries between 2014 and 2020. A standardized criteria and independent assessment were the hallmarks of this process [28]. Case studies were conducted, by the same researchers, using a unified approach examining the case studies in several areas: the innovative initiative, the implementation process, the organization, the founder, the participants, and the relationship with the health system. All 38 case studies were assessed against the following inclusion criteria for this secondary analysis: the initiative was deemed to be a social innovation (as per the inclusion criteria of the TDR case database) with a focus on primary care delivery; a social innovation implemented in a rural or remote area; and a social innovation with an element of active community involvement. Once a short list of cases was attained, one case (best suited to the topic of investigation) was selected from each geographic region. Three case studies were selected for secondary analysis: The Seal of Health Governance, Philippines; the Kaundu Community Health Insurance Initiative, Malawi; and the Model of Integral Healthcare for Rural Areas, Colombia.

Although the country context for each case was significantly different (see Table 1 below), the health systems of these three countries have several similarities: Philippines [29], Malawi [30] and Colombia [31] each made a commitment to work towards achieving Universal Health Coverage; access to primary care provision in rural areas was significantly less than in urban areas; and affordability was a major limitation to health service access [32].

**Table 1:** Country and health system characteristics

Characteristics	Case 1 The Philippines	Case 2 Malawi	Case 3 Colombia
**Population Size[**30**]**	106.6 M	17.5 M	49.6 M
**Rural population[**31**]**	53.1%	83.5%	19.2%
**Population below national poverty line[**32**,**^33^**]**	21.6%	51.5%	26.9% (2018)
**Life Expectancy at birth[**34**]**	71 years	64 years	77.1 years
**Maternal Mortality Ratio[**35**,**^36^**]**	121 (latest data - 2017)	358	83 (2017)
**Total health expenditure** (THE) per capita (PPP) [37]	$371.74 (latest data - 2017)	$115	$1039
**Out of Pocket Expenditure** (% of the population) [38]	53.05%	11.4%	16.3%
**Proportion of population spending more than 10% of household consumption or income on out-of-pocket health care expenditure (%)[39**,**^40^**]**	6.3% (2015)	4.2% (2016)	8.19 (2016)
*Problems in accessing healthcare (distance to health facility) **(% of women)[**41**,**^42^**]**	20.1% (2017)	61.6% (2016)	
*Problems in accessing healthcare (getting money for treatment) **(% of women) [43],[44]**	50.1% (2017)	57.7% (2016)	
**Nurse, midwife density** (per 1000 population) [45]	4.935	0.4 (2018)	1.33 (2018)
**Health System** Structure	Decentralized,	Decentralized	Decentralized
Coverage	A functional national health insurance fund. 94% of the population covered.	No national health insurance but an Essential Healthcare Package delivered at 52% of public health facilities	There is a compulsory national health insurance system. This system consists of contributory and subsidized plans: salaried/independent workers are under contributory plans and subsidized plan for those unable to pay
Health Facilities	40% of health facilities are public. 35% Government hospitals accredited by PhilHealth, 65% of private hospitals accredited. [46]	58% government run health facilities (services are free at point of care) 29% health facilities run by the Christian Health Medical Association (CHAM) [47]	
Commitment to UHC	National Health Insurance Act (2017-2019)	Health Sector Strategic Plan II (2017-2022)	Statutory Health Law (2015)
UCH Effective Coverage Index [48]	55	56	74

### Data collection

Primary case data collection occurred at different time intervals at each of the case implementation sites: January 2018 – February 2019 (Philippines); May – August 2018 (Malawi) and November 2019 – February 2020 (Colombia). Data collection included qualitative interviews and focus groups, participant observation, and organizational and national document reviews, with semi-structured interview guides or focus group guides tailored according to the actors involved (founder; other implementers, beneficiary community members, initiative partners). These interview guides were standardized across countries, for all case studies done as part of the TDR database. Interviews and focus group sessions were conducted in person, at the implementing initiative site (in the rural area). Interviews lasted 30 – 60 minutes each and were recorded and subsequently transcribed. Informal discussions, as occurred during the site visit of the various activities, provided further information. Notes from these discussions and observations from the researcher were captured in study journals.

Actors were selected for participation in each initiative, based on a stakeholder mapping exercise conducted for each initiative, as determined by the actor categories. Where possible, actors not directly associated with each of the case studies, were interviewed to get a broader range of perspectives in challenges, conflicts and influences on social innovation initiative on the country’s health system.

Data were stored on research online password protected databases.

### Analysis

A cross-case analysis was conducted taking a case-based approach by which the integrity of the entire case was retained and then synthesized with emerging patterns arising within each case. Our goal was to explore holistically and advance the understanding of the role of social innovation in extending UHC within rural contexts. The cross-case analysis was conducted with the aid of a simplified version of the conceptual framework  on social innovation as proposed by Cajaiba-Santana [20] (see Fig. 1). The framework, drawing upon neo-institutional theory [33] and structuration theory [34], proposes four dimensions of the social innovation process at different levels of action: the actors or agents responsible for the idea, the new idea, the influencing role of the institutional environment, and the observed changes in the system as a result of the innovation. The process and practices associated with the new idea result in changes which impact on the actors, institutions, and systems. To this framework we add context, as social innovation is contextually dependent and constructed.

### Ethics and other considerations

Researchers involved in this study were drawn from each of the participating universities and independent research agencies including: the University of Malawi, the University of the Philippines, Centro Internacional de Entrenamiento e Investigaciones Médicas (CIDEIM) and the London School of Hygiene and Tropical Medicine (LSHTM). These institutions received a research grant from TDR, the Special Programme for Research and Training in Tropical Disease. Ethics approval for this research was gained from the respective research ethics boards of partners involved in the research: University of the Philippines Research Ethics Commission; Malawi National Commission for Science and Technology, the CIDEIM Research Ethics Board, and the LSHTM Ethics Committee. Social innovation initiatives received no financial compensation for their participation in this study.

## Results

In the sections below, we discuss the cases using the analytic framework. We first describe each ‘new idea,’ which led to the social innovation case being adopted, designed to enhance universal health coverage. We then focus on the agentic actors who were responsible for developing and implementing each ‘new idea’ within their respective context and describe and analyze the enabling and constraining factors inherent in the larger institutional environment within which the innovations were implemented. We conclude by highlighting the effects each social innovation ‘idea’, had not only on the health but also on the community system in each setting.

### New ideas and approaches to extend UHC

Each of the three cases adopted a different and creative approach towards achieving Universal Health Coverage within their localized rural setting. Approaches focused on enhancing access to care, reducing out-of-pocket expenditure, improving quality of care provision, and addressing select social determinants responsible for ill health. Table 2 below provides brief summaries of each of the social innovation initiatives.

**Table 2:** Social Innovation Case Overview

Case	Location	Established	Implementing agency	Problem addressed by the Innovation	Summary of Idea	Innovative Components	Funding Sources	UCH Dimension
Seal of Health Governance	Municipality of Del Carmen, Surigao Island, the Philippines	2012	Office of the Mayor and Municipal Health Department	A geographically isolated and disadvantaged area (GIDA) with a poverty incidence of 58%. Limited health professionals to serve over 20 communities. Persistent poor health indicators especially for material and child health, nutrition, sanitation, family planning.	An inter-village competition and community health leadership program promoting community-initiated health interventions and monitoring by village leaders. Communities are addressing health issues themselves, instead of being solely dependent on limited health providers.	i The training program for leadership in health ii A scorecard that is co-created with community leaders and features a set of health performance indicators and targets iii Awards recognizing good performance and community-based initiatives and innovations for health.	Local government of Del Carmen, Surigao del Norte	• Enhanced service availability. • Financial protection (due to disease avoided)
Kaundu, Community Based Health Insurance	Dedza-East District, Central Region, Malawi	2015	Kaundu Community Health Centre, an affiliate of Christian Health Association of Malawi	29% of Malawian primary health services are provided by the Christian Health Medical Association (CHAM). CHAM health facilities require fee for service. Rural remote communities live below US$1.25/day. The only access the Dedza-East community had to health services is through the CHAM health facility, thus limiting their access to care due to cost barriers.	A community-initiated and managed health insurance scheme, in the context of no functional national health insurance in Malawi.	i. Community management and accountability of the health insurance scheme ii. Membership contributions affordable for a rural population iii. Community sensitization and insurance education	Danish Church Aid, Christian Association of Malawi, Community contributions	• Financial Protection. • Enhanced service access • Service Quality Improvement.
Model of Integral Healthcare for Rural Areas	Sumapaz District, City of Bogota, Colombia	2001	Nazareth Hospital (2001-2015) and Subred Sur (Since 2016).	A post-conflict dispersed rural community, with limited access to care due to the mountainous terrain and related geographic barriers. This farming community experienced health related conditions due to pesticide use.	A multi-disciplinary primary care model, developed through co-learning and co-participation with the community, inclusive of clinical, cultural and environmental health approaches and modalities.	i. Home consultations by a multidisciplinary team, inclusive of indigenous health providers. ii. Indigenous medicine provision along with conventional medical care iii. Agricultural education center delivering training to local farming community iv. Community health network groups on various topics v. Health routes – managed transport and referral process from rural health center to urban hospital	District Health Office of Government of Bogotá City	• Enhanced service access. • Service Quality Improvement. • Financial Protection.

A key principle underlining each of these initiatives was the shift required in prevailing power dynamics associated with health expertise and knowledge, as well as accountability.

"The theme park in public health, which is the scenario where we do the processes of education of recovery of this knowledge of the farmers, of dialogue of knowledge ... really there has been an interaction between the farmer and the health professional, becoming one, with a dialogue of knowledge in which we are equal ... we plant medicinal plants, it is called the medicinal garden, and that is what we want to keep alive this knowledge that the grandparents have" (Model of Integral Healthcare for Rural Areas, Implementer, Sumapaz).

"Our (chief) role is to oversee everything we see how things are going with the committee (insurance committees – community members responsible for community mobilization and day to day implementation of the community insurance)**.** And the committee, when they are working, they are also meant to report back to the chiefs. The chiefs and the community are called and briefed together this is how things are progressing, like this, then we tell them that ok, go and deliver the money to the hospital in agreement with village members" (Kaundu Community Health Insurance, Village Headwoman, Dedza-East).

The social innovation models in Colombia, Malawi and the Philippines all challenged the notion that health expertise and knowledge can only be derived from technical experts. These innovations were grounded in a core belief that community members have valuable knowledge to contribute, based upon indigenous cultural practices and lived experiences. These innovations created a space within the institutional context where this form of knowledge was welcomed and regarded as a resource. Implementation responsibility and accountability for each initiative was given to the community. The Malawi case study provides a strong example of how even financial management and oversight can be delegated to community members. Tasking and trusting the communities with this function shifted the focus of power to them, and as a result enhanced their ownership and sustained their involvement in the initiatives.

### Agents activating agents

Each social innovation was created by a different type of actor, with varying backgrounds in healthcare and from different sectors. In the case of the Philippines, the initiator was the municipal mayor, from the Local Government Unit. Under the Filipino Local Government Code, all responsibility for service delivery, including health service delivery, is devolved to local government level with oversight from the national Department of Health. With no prior background in healthcare, the mayor had the opportunity to be part of a leadership program hosted by a Filipino-based family non-governmental foundation (Zuellig Family Foundation). This program stimulated him to shift his thinking regarding *barangay* captains (local village leaders). Barangay captains oversee each village within a defined geographic area. The mayor saw the opportunity to leverage their local leadership to a greater extent as a way to address a number of suboptimal health indicators. The mayor, wanting to ‘pay forward’ the leadership training he received, established a leadership and governance capacity training and coaching program for the barangay captains. In typical rural municipalities like Del Carmen, these barangay captains, in the governance of health and wellbeing of the community, used to serve as passive implementers of vertical health programs or merely report data. The opportunity for barangay captains to extend their leadership capacity, and hence their autonomy, was extended through the implementation of the inter-village competition based on co-created scorecards between the villages and the local government office. In this way, barangay captains, in collaboration with their communities, had to propose and develop their own ideas to improve the scorecard indicators. The resources required for this new initiative were supported by the mayor through the municipal budget.

"There is a necessity to create more leaders to share your vision and to do the work with you for the people. That is essentially what we have been doing through bridging leadership and the Seal of Health Governance: empowering more health stakeholders, empowering more health leaders that share our vision of a healthier community for Del Carmen" (Seal of Health Governance, Senior Leader, Del Carmen).

In the case from Malawi, the initiator was a parliamentary health leader, a nurse-midwife by training. Within her area of jurisdiction, the Dedza-East district in Central Malawi, she engaged local health stakeholders to assist her to realize the idea of a community-owned micro health insurance program. As the majority of health services in the area are provided through the Christian Health Association of Malawi (CHAM), this agency drew upon its technical, training and administrative expertise to assist the development of the community-owned insurance and health needs mapping. The agency leveraged its existing relationships with frontline staff, community members and local leaders in order to support these processes. Start-up funds were provided through a micro-finance model (Village Savings and Loans) by CHAM, which provide small business loans to groups of community members to establish local enterprises. Through the profit generated, the community had the initial funds required to invest in a community-based insurance scheme. The day-to-day management of the scheme is undertaken through involving community members and local staff of the primary health care center. Health Insurance Committees were established, comprised of community health workers, traditional leaders (chiefs and village headman) and community members. These committees took responsibility for educating the community, mobilizing member enrolment, registering new insurance members, collecting monthly membership premiums, and providing updates to the traditional leaders. Monthly premiums are deposited at the local health center, and committee members track each community’s trend on expenditure and service use. Community members volunteer their time to support health week, outreach activities and delivery of services. In addition, they conduct patient satisfaction surveys and provide feedback to the health center on the type and appropriateness of services they receive.

In the third social innovation case from Colombia, the initiative was developed by a small group of frontline health workers from the local primary health center. The health center adopted a systematic bottom-up approach to determine the health needs and the environmental risks related to health of the local farming community. Community members were integral in this process as it gave them the opportunity to share their challenges, needs and opportunities, but also to contribute their own cultural and social knowledge of the region and daily life. Additional epidemiological and cartographic data on the environment were also collected. This combination of methods, data and knowledge assisted in providing a comprehensive understanding of the complexity of people’s lives, interpreting and prioritizing health needs in a contextually sensitive manner. This process laid the groundwork to inform the further development of different programmatic components or strategies, in close partnership with the farming community. In addition, the model placed strong emphasis on promoting education and leadership in the population, through different strategies. This led to the development of health leaders with knowledge of their rights, who were able to guide their communities to adopt practices that are regarded to improve health, and to actively participate in identifying the needs and creation of solutions:

"In order to generate impacts and that families really believe us ... has been precisely because their needs have been identified, and they have been participants with influential participation in the whole process, we have never managed to impose things that occur to us, but always from the reading of what are those needs that the farmer has" (Model of Integral Healthcare for Rural Area, Implementer, Sumapaz).

### Institutional enablers and constraints

To understand social innovations implemented within rural settings, a deeper insight is needed of the broader institutional and health system context within which social innovations are brought into being. Noteworthy factors within the institutional context contributed to enabling or constraining aspects of each of these three cases.

In all three countries, the health system was decentralized. Within this institutional framework, the prescribed devolution of power from the top to the lower levels of the health system enabled actors such as the Mayor of Del Carmen (Philippines), the Christian Medical Health Association (CHAM, Malawi), and the local hospital team (Colombia) to have implementation authority. In the Colombian case, the innovation was also supported by a policy framework encouraging social participation in Bogotá City, and this in turn motivated the health team to try new forms of community-based health interventions in which the community was included in both development and delivery. In Malawi, 29% of health service provision is delivered by CHAM [26], of which the primary care facility is one such facility. In each of the cases, the seniority position of the social innovators contributed to moving from idea to implementation stage.

Crucial also were arrangements made by institutions to enable increased representation of community members and other stakeholders. In the Philippines, the law mandates the creation of local special committees – such as the Local Health Board – to inform policymaking. In the case study, the municipal government expanded the Local Health Board to include village leaders and civil society actors. In Malawi, the Health Facility Advisory Committee was reorganized to play an important accountability role for health services provided by the respective facility. The existence of formalized community accountability structures supported the implementation of the community-based insurance scheme.

A second enabling factor was the willingness of organizations and agencies across the health system, and from other sectors, to participate. A key principle of the Colombian innovation was to ‘build upon that which has been built’, and in so doing, giving continuity to existing processes. Simply by linking actors, new resources were unlocked. Intersectoral and interinstitutional collaboration was core to the successful implementation of this initiative. Partners in this initiative included organizations from across all health system levels (primary, secondary and tertiary care) as well as universities and environmental agencies. In this way, the model’s potential impact was extended more widely, new holistic knowledge on health was generated, and social determinants responsible for ill health could be addressed in a harmonized way. Similarly, in the case of the Philippines, the Mayor and Local Government continued to receive support from the Zuellig Family Foundation in the first round of implementation. This initiative also enabled greater collaboration between local government structures and the Municipal Health Office as they shared implementation duties. This provided local elected officials with a deeper understanding of the health system, leading to their increased buy-in to support health programs. In the Malawian social innovation case, extensive effort was undertaken to ensure all health system structures were engaged in the initiative: these included the district health management team, the district executive committee, the area and village development committees, and the health facility advisory committee. The broad base of collaboration within the institutional environment served to unlock new resources that were required to implement these initiatives.

Barriers faced by these social innovations included initial resistance from local actors and communities lacking the knowledge or understanding of the subject area. In the Malawi case, insurance was a new concept for the rural community and significant investment was made in community education. In the Philippines, the community did not have sufficient understanding of health metrics, and as part of the initiative, they were educated to understand the implication of their own local health indicators and to contribute to improving these indicators according to set targets, without significant monetary incentives for participation. Further, efficient and timely monitoring of programs remained in dispersed geographic settings as found in Dedza in Malawi and Sumapaz in Colombia. Poor mobile phone coverage as well as limited internet and electricity made basic reporting functions more cumbersome. In the Malawi case study, all data collection remained paper-based and dependent on volunteers covering vast distances to collect data from community members.

In Colombia and the Philippines, the initiatives are dependent on the current political leadership, so potentially limiting the future of these initiatives. In Colombia, the initiative is influenced by political changes within the greater Bogotá district and is entirely dependent on the municipal budgetary allowance. In the Philippines, the momentum for the initiative comes from the Mayor and Municipal Health Officer, and both these positions are subject to change.

As in all new or creative initiatives, resources are essential. Although sustained resources (financial or in-kind) were required by all three initiatives, ways were identified to attract resources vis a vis private sector partners, community involvement, locally established non-profit foundations, community contributions (especially time investment). In the Colombian case resources for the initiative came from multiple sources: funding came from the Bogotá city district health office; an award from the City’s botanical garden provided resources for infrastructure in agricultural education park, the National University provided community training on food security income generation projects, the Ministry of Science provided research support to the expert researchers from the university and the implementing team. Most importantly, community volunteers provided their time to run and manage the community networks.

### Effects on the community system

Each of the three cases resulted in a marked improvement in local health indicators. Among the most significant were indicators on maternal and child health. In the Philippines case, four out of 20 participating villages reduced malnutrition identified in infants and children to zero; facility-based deliveries in the municipality increased from 89 to 98%, so halving the infant mortality rate [35]. In Malawi, the social innovation initiative resulted in a ten-fold increase in facility-based deliveries, service utilization by children under-5 increased by 60%, and there was a near doubling of adults presenting to outpatient services (September 2014 to September 2017) [36, 37]. The Colombia innovation was able to achieve 100% service coverage for its population, reduced maternal mortality and under-five mortality, and malnutrition down to zero in the last three consecutive years (2016, 2017, 2018, most recent data) [38].

An additional observed effect was an improvement in the motivation and attitudes of local health center staff in each of these initiatives. Staff generally expressed their pleasure and willingness in participating in these innovations:

"It helps a lot especially now that we have received an award. It’s like we are challenged or pressured to either maintain the initiative or do even better. Perseverance and determination are really key. Serve from your heart so that the people will see and appreciate it" (Seal of Health Governance, Nurse, Del Carmen).

"At times in the past when community members see us (health workers) coming, they would run away, thinking we are coming to collect debts on services provided. Now with this insurance, communities see and recognize our role as part of the process for good health" (Kaundu Community Health Insurance, Head Nurse, Dedza-East).

In the Colombian social innovation case, through the home health visits, health workers expressed their satisfaction in being able to deepen relationships with their community and to get to know community members as whole people, beyond the diseases that brought them to seek clinical advice. The increased level of engagement of frontline staff with community members and the creation of more equal and open channels for dialogue between them led to improvements in staff motivation and attitudes; this, they suggested, was likely a contributing factor enhancing service quality in these areas:

*“*the recognition that they [ the Sumapaz community] also make us, so let’s say we [health workers] are one more family*”* (Model of Integral Healthcare for Rural Areas, Implementer, Sumapaz).

The most significant influence of the three social innovation cases were on strengthening the community system, in health but also in areas such as the environment, economic development and gender equality. In the Philippines, local villagers proposed the development of ceramic toilet bowls as a way to improve the indicators around sanitation. This micro-initiative was so successful that it became a source of income for the local community, as once their own demand was met, they were able to sell toilet bowls at an affordable price to other villagers. Similarly, in Malawi, the seed capital provided for new business development, as a way for villagers to earn the income required to join the insurance scheme, supported local economic development. In Colombia, some of the most important actions related to food security, nutrition and environmental education were aimed at women’s groups, so that while improving health, women’s empowerment was also supported:

“Community members in one of the catchment villages rented a rice scheme and all the proceeds after selling were deposited at the health facility to ensure that each member was part of the insurance” (Kaundu Community Health Insurance, Health Surveillance Assistant, Dedza-East).

In Colombia, community members played an important role in various community health networks and participated in the maintenance of the agricultural training center. Ten community networks were created to train health promoter community leaders on different topics related to integrated health and differential care. Each of these networks is made up of a minimum of eight people who maintain their participation during the 4-year implementation cycles. According to needs identified by the community, theoretical-practical training is provided in hands-on workshops in a public health park on specific topics unique to the network such as the promotion of home vegetable gardens, organic waste management, healthy eating practices, healthy work, good agricultural practices, and the decreased use of and control in the use of pesticides.

## Discussion

**Fig. 2:**
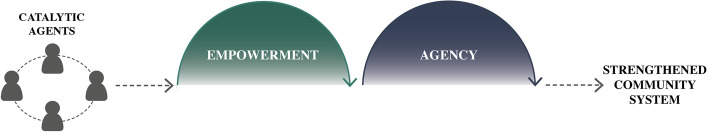
Social innovation process at community level

The three cases studied are social innovations in health based on the development of community capacities to achieve health. This is understood as the process by which individuals, groups, organizations, institutions and societies increase their abilities [36], through different means: in the Philippines, the focus was on building leadership capacity among barangay captains; in Colombia, on co-learning through knowledge sharing between professionals and the community; and in Malawi on enhancing community implementation and accountability capacity. Below we seek to describe the process that unfolded that led to the success of these social innovation initiatives in improving access and quality of health services for the local rural and vulnerable population (Fig. 2).

### Institutionally embedded agents as catalysts of community social innovation

The three cases illustrated how a single actor or small group of actors played a catalytic role as institutional entrepreneurs in initiating a process of social innovation [39]. These actors drew on their embedded knowledge and influence within the institutional context to create a space for innovation, in an otherwise very structured and bureaucratic environment. By playing the role of ‘boundary-spanners’ [40], the spaces created by these institutional contexts, these actors facilitated interaction with other stakeholders across different levels of the health system (communities to authorities) and across sectors. Each of these initiatives were made possible by a multilevel inter-institutional network and cross-sectoral network. In operating in this unique role, these actors were able to effectively engage with communities and at political levels within their countries. They were successful in unlocking dormant system resources which, once pooled, enabled implementation to occur despite the resource constraints of each setting.

The founders of each of these initiatives had a relative level of authority over a specific sphere of influence and instead of following traditional public health lines of top-down, vertically imposed authority flows, they each operated with a more distributed and shared leadership approach – acknolwdging the authority and agency residing within community members and working in collaboration with them.

### Social innovation participation as a means of community empowerment

In each of the three social innovation cases communities were involved along the full continuum – from initiative design, implementation, and monitoring. The innovation design process was heavily influenced by a co-learning and knowledge exchange process. The institutions offered new ideas and platforms of solutions, at the same time as communities shared their own embedded cultural and social knowledge of daily life within the defined areas. From the beginning, a power shift took place, away from an initiative being imposed in a top-down manner by ‘external experts’ to one that was co-created and co-owned by the community. This respectful participation of community members and the value placed on their knowledge and skills, albeit non-technical or non-academic, was key to community empowerment.

Community members were not only given a role as participants but also as leaders. This was achieved through recognizing their existing leadership (e.g. Barangay Captains or Village Chief) and by providing opportunities for education such that people had the knowledge to make informed decisions on behalf of their community – training on health insurance in Malawi, agricultural training in Colombia, and leadership training in Philippines. The communities became the leading actors in the implementation, while institutions assumed a support role for the communities by providing technical guidance and unlocking resources in other sectors.

### Agentic communities taking action for health

Sen defines agency as ‘what a person is free to do and achieve in pursuit of whatever goals or values he or she regard as important’ [41]. As also described by Tiwari [25], social innovation plays an important role as a ‘conversion factor’ to help communities go from their aspired goals to achieved goals. In each of the cases we observed how the social innovation process was key to facilitating a shift in power (by giving communities an opportunity for participation). The intrinsic value of respect for the community within this process, by regarding community knowledge as at an equal level to those of technical experts, led to empowerment being concretized in the form of agency. This agency enabled community members to play an active role in the implementation of these initiatives, and in turn this was a strong contributor to the sustainability of each social innovation and to the achievement of sustained improvement in health indicators. In addition, the raised levels of agency in the community supported greater social accountability of the local health services. To date, several mechanisms have been considered to enhance social accountability, including community action groups, social audits, score cards, and so on [42], but social innovation appears to be a mechanism to achieve greater social accountability within communities.

### Achieving universal health coverage

The case studies illustrate how social innovation as an approach can be useful to achieve changes in the social and institutional system, and to build sustainability. These creative solutions were not only based upon actualized community needs, but also took into consideration a much broader perspective on health – one inclusive of social, environmental, and cultural factors. Not only did community health indicators in each setting improve, but simultaneously greater capacity was built for community leadership, economic development, and environmental stewardship. By raising community empowerment and agency, these communities were able to apply the skills and knowledge they received to solve other social challenges in their area, and to nurture their own personal and career development. In particular, the Colombian case illustrates how a strengthened community system was a key factor to support the sustainability of the social innovation for 20 years beyond the short duration of funding or political cycles. Beyond supporting the progress towards the Sustainable Development Goal 3 (Universal Health Coverage), these social innovation initiatives also supported progress towards Goal 1 (poverty reduction), Goal 2 (hunger reduction), Goal 5 (gender equality), Goal 12 (responsible consumption and production environment) and Goal 13 (climate action).

### Limitations and future research

This study is an initial exploration of the role of social innovation on community health systems. We recognise the limitations of the small comparative sample. Each of the case studies represents a distinct geographic context, and there would be benefit in enhancing the contextual analysis and implications of each setting. All case studies included in the TDR database, from which this sample was purposefully selected, were included because of them being ‘cases of successes. Thus, this study is limited in that it did not study examples of initiatives which were unsuccessful, thus missing out on the learning that could be gained from failed cases.

Future research would benefit in developing a set of indicators by which the success of social innovations in strengthening community health systems could be measured. Further opportunities for future research also entail: a quantitative analysis complementing qualitative exploratory work; deeper investigation of the power structures and dynamics at play, both at an indivual and institutional level and longitudinal research to better understanding the evolving processes over time.

## Conclusion

Despite the value placed on the role and participation of communities in the achievement of Universal Health Coverage, there is limited practical guidance available on how this can be achieved and sustained. This study found that three social innovation models were based on the development of community capacities to achieve health through community co-learning, leadership, and accountability. Through a multi-country cross-case analysis, we have illustrated how three creative social innovation approaches have been successful in enhancing access and equity of health services for rural and vulnerable populations. Underpinning each of these processes were a series of practical steps and actions that supported community participation across the full continuum of initiative development, implementation, and design. These processes facilitated empowerment and increased agency among the community. These in turn supported sustained strengthening of the community system and the achievement of community goals, in the domain of health and beyond.

## Data Availability

The data used for this article is available from the corresponding author on reasonable request.

## Abbreviations

UHC: Universal Health Coverage
LMICs: Low and middle-income countries
SDG: Sustainable Development Goals
TDR: The Special Programme for Research and Training in Tropical Diseases, cosponsored by the United Nations Children’s Fund, the United Nations Development Programme, the World Bank and the World Health Organization.
CIDEIM: Centro Internacional de Entrenamiento e Investigaciones Medicas
CHAM: Christian Health Association of Malawi
